# Using the 16PF to Test the Differentiation of Personality by Intelligence Hypothesis

**DOI:** 10.3390/jintelligence8010012

**Published:** 2020-03-10

**Authors:** Julie Aitken Schermer, Georg Krammer, Richard D. Goffin, Michael D. Biderman

**Affiliations:** 1 Management and Organizational Studies, Faculty of Social Science, The University of Western Ontario, London, ON N6A 5C2, Canada; 2 Institute of Practical Education and Practitioner Research, University College of Teacher Education Styria, 8010 Graz, Austria; Georg.Krammer@phst.at; 3 Department of Psychology, Faculty of Social Science, The University of Western Ontario, London, ON N6A 5C2, Canada; goffin@uwo.ca; 4 Department of Psychology, Professor Emeritus, The University of Tennessee at Chattanooga, Chattanooga, TN 37403, USA; mdbiderman@gmail.com

**Keywords:** differentiation, personality, intelligence, 16PF

## Abstract

The differentiation of personality by intelligence hypothesis suggests that there will be greater individual differences in personality traits for those individuals who are more intelligent. Conversely, less intelligent individuals will be more similar to each other in their personality traits. The hypothesis was tested with a large sample of managerial job candidates who completed an omnibus personality measure with 16 scales and five intelligence measures (used to generate an intelligence *g*-factor). Based on the *g*-factor composite, the sample was split using the median to conduct factor analyses within each half. A five-factor model was tested for both the lower and higher intelligence halves and were found to have configural invariance but not metric or scalar invariance. In general, the results provide little support for the differentiation hypothesis as there was no clear and consistent pattern of lower inter-scale correlations for the more intelligent individuals.

## 1. Introduction

Research testing the differentiation of personality by intelligence hypothesis has had varied results. Following the finding of greater differentiation in cognitive ability measures for those individuals who were more intelligent (Spearman 1927; see also Deary et al. 1996; Deary and Pagliari 1991; Detterman and Daniel 1989; Lynn 1992), Brand et al. (1994) suggested that the variability of personality measures may also be higher for those higher in intelligence. In a similar but much earlier vein, McDougall’s (1929) observation that humans’ characteristics are formed to serve the actions that individuals seek to complete suggests that the personality traits of more intelligent individuals might naturally be more differentiated as a means of achieving more complex goals. If the differentiation of personality by intelligence hypothesis is found to have some generality, it suggests that intelligence may interact with personality in ways that have not been sufficiently considered in the literature. This interaction may have important implications for the measurement of personality if measures are effected by the intelligence level of the test-taker. In the following discussion, the present study adds to the literature by testing the differentiation of personality by intelligence hypothesis in a sample of candidates for mid-level managerial positions. Although there may be a restriction in the personality test score range, because the sample were applicants and presumably presenting themselves in the most favourable manner, if the pattern of results do suggest more personality variability for those higher in intelligence, this finding would support the differentiation hypothesis within a high-stakes setting.

Shure and Rogers (1963) were possibly the first to demonstrate that intelligence influences the structure of personality. Concerned that the factor structure of personality measures may not be consistent across different cognitive ability levels, the factor structure of the California Psychological Inventory (CPI; Gough 1957) was examined for three groups of students who were categorized as low, moderate, or high in intelligence. Utilizing various methods of factor extraction for each ability group, the number of factors varied greatly both across factor extraction methods and ability groups. Of interest to the differentiation of personality by intelligence hypothesis, the factor loadings differed across ability level groups. For example, the sum of the squared factor loadings for a “personal integrity and mental health” factor was highest in the higher ability group, followed by the middle group, and was 2/3 the amount of the higher ability group in the lower ability group. Shure and Rogers (1963) concluded that if samples of people are drawn from different ability groups, then the definition of personality factors, based on the factor loadings, could change even if the same battery of personality scales is used for each sample.

The results reported by Shure and Rogers (1963) do lend some support to the idea that intelligence may influence the structure of personality traits but are not direct tests of Brand et al.’s (1994) hypothesis that individuals higher in intelligence will show greater differentiation in personality. In a test of the differentiation hypothesis, Austin et al. (2000) found that the variance in psychopathology measures differed for individuals at different cognitive ability levels, suggesting that intelligence may be related to different clinical traits depending on the individual’s ability level. Based on a median split of an intelligence score generated from 10 ability scales (five verbal and five performance), Harris et al. (2005) tested both personality scale scores and factor scores between higher and lower intelligence groups based on the 20 personality dimensions from the Personality Research Form (Jackson 1989). For the majority of the comparisons at the scale and factor levels, there was greater variability in the higher intelligence group. Subsequently, Harris et al. (2006) tested the variability of personality, using the Big Five personality dimensions, in intelligence level groups and reported that the differentiation hypothesis earned some support in a sample of adults but not in a sample of adolescents.

Mõttus et al. (2007) reported that although the structure of personality factors, based on the Big Five personality dimensions, did not change across two ability-level groups, the correlations between the personality scales was lower in the higher ability group compared to the lower ability group. Murray et al. (2016) tested the differentiation hypothesis with the 16PF and found greater variability in a higher ability group for anxiety but not for extraversion, self-control, independence or toughmindedness. De Fruyt et al. (2006), based on a large sample of job candidates, reported that although the factorial structure of the personality scales, based on the Big Five personality model, stayed the same across intelligence groups, variance for openness, neuroticism, and extraversion were slightly higher for the higher intelligence group. In general, these studies, which have tested the differentiation hypothesis, have provided limited support for the differentiation hypothesis with some finding greater variability in personality traits for individuals higher in intelligence and others failing to find an effect.

One possible limitation to some of the studies reviewed above was the use of single measures of intelligence. For example, Murray et al. (2016) utilized the reasoning scale from the 16PF as a proxy measure of intelligence. Similarly, De Fruyt et al. (2006) utilized a single performance intelligence measure. The present study has the advantage of having five cognitive ability measures that can be used to generate a composite *g*-factor, similar to the study by Harris et al. (2005).

In the present study, we test the differentiation of personality by intelligence hypothesis with two groups, higher and lower intelligence groups, based on a median split along a *g*-factor created from five intelligence scales. The median split was used in accordance with previous studies addressing the differentiation of personality by intelligence hypothesis. The aggregation of the five intelligence scales overcomes the limitation of previous studies, which often only addressed selected aspects of intelligence. As stated above, the sample were candidates for mid-level managerial positions. As part of their assessment, the candidates completed both personality and intelligence measures. The intelligence groups are assessed for possible differences in the variance and structure of the personality measures. If the differentiation of personality by intelligence hypothesis is supported, then we should find greater variance in personality for the higher intelligence group and lower inter-scale correlations resulting in different factor structures for each group using the same extraction and rotation methods.

## 2. Method

### 2.1. Sample and Procedure

Participants were 1198 (10% women) candidates for mid-level managerial positions in a large Canadian forestry products company. As part of the candidates’ assessment, personality and intelligence test scores were collected. The mean age was 37 (*SD* = 7.5), with a range of 21 to 59 years. The highest level of education data were available for 252 participants, of whom, 1.2% reported graduate degrees, 42.9% reported university degrees, 40.9% reported college diplomas, 4.4% reported “some” post-secondary education, 9.9% attained high school graduation, and 0.8% reported “some” high school education. Portions of the data have been analyzed in earlier publications, however, none of these previous publications addressed the current research question. A list of these publications is available from the third author.

### 2.2. Measures

#### 2.2.1. Sixteen Personality Factors (16PF)

Form A of the fourth edition of the 16PF was administered (Cattell et al. 1970). This scale includes 16 primary trait scales (warmth, reasoning, emotional stability, dominance, liveliness, rule conscious, social boldness, sensitivity, vigilance, abstractness, privateness, apprehension, open to change, self-reliance, perfection, and tension) which have a long history of on-going development and refinement. Although the data available did not include item level responses (to generate internal consistency estimates), the 16PF has been reported to have good reliability (Cattell et al. 1970).

#### 2.2.2. Intelligence

Intelligence was measured using the Employee Aptitude Survey (EAS; Ruch et al. 1994), which includes verbal comprehension, verbal reasoning, numerical ability, numerical reasoning, and symbolic reasoning. Using these scales, a general intelligence (*g*) score, generated using a factor analysis of the five scales, was computed (see below).

#### 2.2.3. Assessment

The administration of the 16PF and EAS were supervised by a registered psychologist as part of a standardized Assessment Center consisting of a variety of managerial evaluations (see Cascio and Aguinis 2011). The assessment required two days of each candidates’ time. The individuals were evaluated in groups of five. The 16PF and the EAS were administered on the second day of the assessment and the order of administration was randomized. The participants completed the evaluations for operational purposes within the forestry products company. They were informed that their results would be used in their assessments for managerial positions and in research on the assessments. After their operational use by the organization, the data were obtained from the registered psychologist who ran the Assessment Center as archival data that had been completely anonymized. The Chair of Western University’s Non-Medical Research Ethics board approved the use of these archival data for research purposes.

#### 2.2.4. Data Analysis

The first step in data analysis was the computation of the general intelligence factor. This variable was used to separate the sample into lower and higher intelligence groups based on a median split along the general intelligence factor. Within each group, tests of mean differences (*t*-tests) and variance differences (*F*-tests) were computed to examine if the higher intelligence group had more variability in the scale scores than the lower intelligence group. During the following stage of this analysis, the inter-scale correlations and factor structure of the 16 personality scales were examined within each intelligence group. Then, the tests of invariance were conducted on the resulting factor structure for each intelligence group.

If the factor structure of the 16PF is invariant across the two intelligence groups, then it would be expected that factor analyses of the 16 scale scores would yield essentially identical results in the two groups. A sequence of tests designed to assess invariance of factor solutions between the low and high intelligence groups is conducted. The sequence first assesses configural invariance—that is, the similarity of factors across the two groups, then metric invariance—the equality of loadings across the groups, and, finally, scalar invariance—that is, the equality of intercepts across the groups. Please note that testing the differentiation of personality by intelligence in the measurement invariance context is only a straightforward matter if measurement invariance is given. Interpretations of violated measurement invariance may be obscured for two reasons: first, the differentiation of personality by intelligence may simultaneously cause violations on different levels of measurement invariance, which can be difficult to separate meticulously; second, it relies on the assumption of a certain amount of homogeneity within heterogeneous intelligence groups, which may not be testable due to a lack of sufficient sample sizes in high levels of intelligence.

To test configural equivalence, the same factor model is applied the whole sample, and then to the lower intelligence group and to the higher intelligence group, allowing all parameters to be freely estimated separately in each group. Evidence for configural invariance is indicated if the results suggest roughly equivalent goodness-of-fits between the two groups and a rough comparability of the goodness-of-fit measures for the two applications combined compared to the goodness-of-fit of the model applied to the whole sample.

The second step in the process involves determining the invariance of loadings across the two groups. It involves comparing goodness-of-fit measures for the analyses applied to the two groups, allowing loadings to be unequal across groups, with analyses applied to the two groups requiring corresponding loadings in each group to be equal. In this comparison, intercepts are allowed to differ across groups. Small differences in the goodness-of-fit measures in the two analyses suggest metric invariance. Large differences suggest that the model applied to one of the intelligence groups requires different loading patterns than does the model applied to the other intelligence group.

The third step involves comparing applications of models with equal intercepts across groups with models in which intercepts are allowed to be unequal. Whether loadings are estimated differently, or restricted to be equal across groups, depends on the result of the analyses in the second step described above. A small difference in goodness-of-fit of models with intercepts constrained to be equal, compared to models with unequal intercepts across groups, supports scalar invariance. A large difference suggests that the pattern of intercepts varies from one group to the other.

Two goodness-of-fit measures are employed for the comparisons. The first is the common chi-square goodness-of-fit measure. Specifically, a chi-square difference measure is computed for the second and third steps. A nonsignificant chi-square in those comparisons would represent invariance across groups for that step. The second was the Comparative Fit Index (CFI). Although there are no statistical comparisons for the CFI measure, for the comparisons here, a criterion value of 0.01 is used, following recommendations by Cheung and Rensvold (2002) and Ock et al. (2019).

## 3. Results

### 3.1. Intelligence Factor

Following principal axis factoring (PAF) extraction, a general intelligence, or *g* factor was created via the first factor. This factor accounted for 34.33% of the variance. A *g* factor score was calculated for each participant based on a weighted linear aggregate with the factor weights of 0.54 for verbal comprehension, 0.70 for verbal reasoning, 0.74 for numerical abilities, 0.75 for numerical reasoning, and 0.72 for symbolic reasoning used in the computation of the factor score.

### 3.2. Median Split

As a means of testing the differentiation hypothesis, a median split of the sample was conducted, based on the composite intelligence factor, to examine the scale variance differences (Levene’s *F*-test). Mean differences (*t*-test) were also computed and reported for completeness. Listed in Table 1 are the tests of scale variability. None of the *F*-values were significant, failing to support the idea that there may be greater variability in scale scores for the higher intelligence half. Significant mean differences in personality were found. The higher intelligence half scored higher on reasoning, abstractness, open to change, and self-reliance. The lower intelligent half scored higher on rule consciousness and privateness.

**Figure 1:**
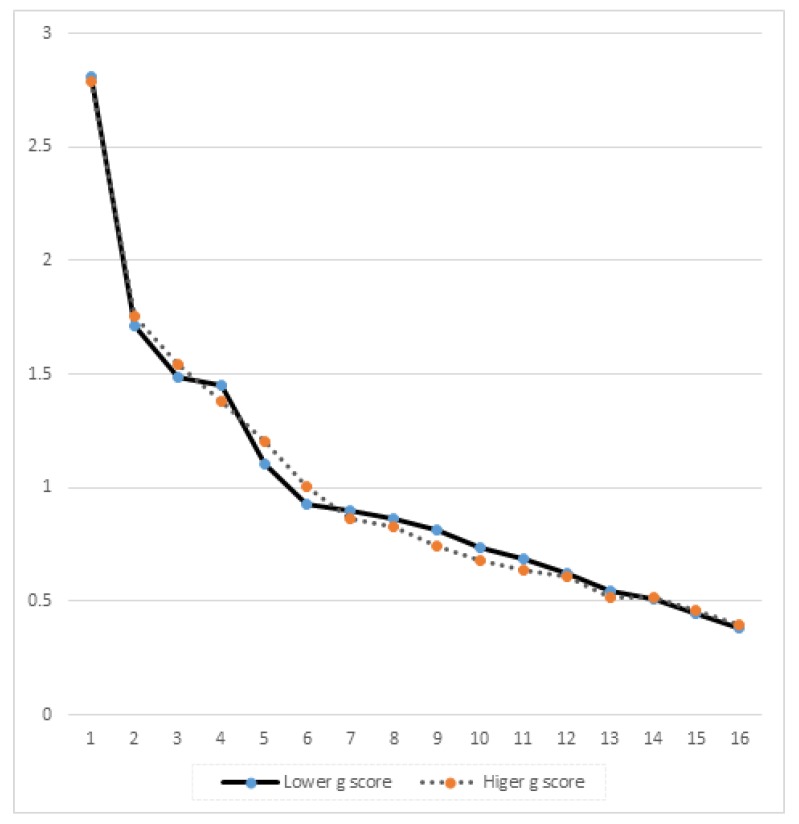
Scree plots for the lower and higher *g*-scoring groups.

For each intelligence half, the 16PF scales were factor analyzed using PAF with direct oblimin rotation. For the lower intelligence half, the Kaiser-Meyer-Olkin (KMO) value (the squared correlations divided by the aggregate of the squared correlations and partial correlations) was 0.680. The KMO value for the higher intelligence half was 0.684, tentatively suggesting that the scales have slightly higher inter-correlations in the higher intelligence group. Figure 1 provides the scree plots of the lower intelligence half (solid line) compared to the higher intelligence group (dashed line). The plot demonstrates a clearer elbow for the lower intelligence group. Using the eigenvalue greater than unity criteria, five factors would be extracted for the lower half but six factors would be suggested for the higher intelligence half. For comparison purposes, five factors were extracted from the higher intelligence half. These five factors accounted for 53.52% of the variance in the lower intelligence group and 54.24% in the higher intelligence group. Following oblimin rotation (see Table 2 for the pattern loadings), the sum of the squared factor loadings was computed (see bottom row of Table 2). For only three of the five factors, the sum of the squared factor loadings was higher in the higher intelligence group than for the lower intelligence group. The individual factor loadings are fairly consistent for each factor with a few exceptions. For example, for self-reliance on factor I and emotional stability on factor II, there is a much larger negative loading for the lower intelligence half than for the higher intelligence half. In contrast, abstractness has a larger loading on factor V for the higher intelligence group than for the lower intelligence group. The mean absolute inter-factor correlations were computed for each half. For the higher intelligence group, the average was 0.101, just slightly lower than the 0.107 for the lower intelligence group. These comparisons provide mixed support for the differentiation hypothesis as although the KMO value and percent variance accounted for suggest greater inter-scale similarity in the higher intelligence half, the more intelligent group’s eigenvalues suggested an additional factor (compared to the lower intelligence half).

### 3.3. Testing Invariance

The first step in the three-step invariance test was the test of configural invariance. A five-factor exploratory factor solution, based on previous research (Schermer and Goffin 2018) was applied to the complete sample of respondents using the Mplus (Muthén and Muthén 1998–2017) ESEM procedure with a GEOMIN rotation, yielding χ^2^(50) = 148.88, CFI = 0.962, RMSEA = 0.041, and SRMR = 0.020. Next, two five-factors exploratory factor analyses (EFAs) were applied—one to the lower intelligence group and the other to the higher intelligence group. All parameters in each group were estimated without constraints across groups. The results for the two analyses combined with freely estimated loadings and intercepts across groups were χ^2^(100) = 199.43, CFI = 0.962, RMSEA = 0.041, and SRMR = 0.025. The fit of the model in the two groups separately was roughly comparable to the fit treating all respondents as a single group, suggesting configural invariance across the two groups.

The second step, assessing metric invariance, involved comparing the model fit when loadings were constrained to equality across groups versus the model allowing all loadings to vary between groups. The results from the model with freely estimated intercepts across groups but restricting loadings to be equal were χ^2^(155) = 280.591, CFI = 0.952, RMSEA = 0.037, and SRMR = 0.034. Comparing the two goodness-of-fit measures for invariance testing from this model, to those from the previous model, yielded a chi-square difference of 81.16 with 55 df, *p* = 0.01, and a CFI difference of 0.01. The difference in fit slightly exceeded the criteria for invariance, suggesting the existence of metric invariance across the two groups.

The final step involved applying a model in which intercepts were restricted to be equal across groups. Because the results of the previous step suggested that loadings differed across groups, the chi-square and CFI of this model were compared to corresponding values from the second analysis in which both loadings and intercepts varied across groups. For the model with freely estimated loadings but equal intercepts, the goodness-of-fit values were χ^2^(116) = 320.603, CFI = 0.922, RMSEA = 0.054, and SRMR = 0.043. The model comparison yielded a chi-square difference of 121.17 with df = 16, *p* < 0.001, and a CFI difference = 0.04, exceeding the 0.01 criterion. Both differences suggest that the intercepts were not invariant across the two groups.

In summary, these results show that the responses of the lower intelligence and higher intelligence groups to the 16PF exhibited configural invariance, suggesting that the number of factors are the same in the two groups. The two groups did not exhibit metric invariance or scalar invariance, however. Loading patterns and intercept patterns differed between the groups.

## 4. Discussion

The hypothesis of the differentiation of personality by intelligence suggests that there is greater between-trait variability for more intelligent individuals. We tested this hypothesis by examining the variance and exploratory factor analysis of the 16PF traits between groups differing on general mental ability in a sample of managerial job candidates. The pattern of results provided little support for the differentiation hypothesis. The differences between the higher and lower intelligence groups did not reach statistical significance for the tests of the variance values. The median split pattern provided some support for the hypothesis with greater variability in the higher intelligence half and with the suggestion of an additional factor in the scree plot and Eigenvalues, and slightly lower mean absolute inter-factor correlations. In contrast, the higher intelligence half had a higher KMO value and the extracted five factors accounted for more variance than for the lower intelligence half. In addition, for the five factors, the sum of the squared factor loadings was slightly higher for the higher intelligence half for three of the five factors. The test of invariance of the two five-factor solutions suggested that the loadings and intercepts differed between the two intelligence groups.

The present sample consisted of managerial candidates and is therefore somewhat similar to the sample of job applicants assessed by De Fruyt et al. (2006). Both samples represent real world assessment situations and present a high-stakes condition for the participants. De Fruyt et al. (2006) reported that their measure of the Big Five personality factors remained consistent for those higher in intelligence compared to those scoring lower in intelligence. In contrast, the higher intelligence half in the present sample suggested possibly one more factor than the lower intelligence half using the 16PF. How the factor structure might differ across intelligence for other personality measures is an area which requires further research.

The present sample is also similar to the study by Murray et al. (2016) in that the 16PF was analyzed. Murray et al. (2016) divided their sample based on responses to the 16PF reasoning scale as a proxy measure of intelligence. Although reasoning has been found to correlate 0.44 to 0.57 with intelligence scales (Abel and Brown 1998), the reasoning score in the present study only correlated 0.30 with the *g*-factor composite (based on five ability measures). How the 16PF items might respond based on a *g*-factor from other multiple ability measures is another area requiring further research. One advantage in the Murray et al. (2016) study was the availability of item responses. Unfortunately, the present study was limited in that only scale totals were available.

Austin et al. (2000) stated that the differentiation of personality by intelligence hypothesis reflects the finding that, “the more able are more variable on the trait dimension” (p. 407). Recently, Schermer et al. (2020) demonstrated that individuals higher in intelligence responded to a Big Five personality scale more consistently (higher internal reliability values), had larger standard deviations, and greater scale variances, supporting the suggestion by Austin et al. (2000; see also Navarro-González et al. 2018). One method that could be utilized in future studies testing the differentiation hypothesis could be to ask individuals to think aloud while completing personality items. If those who are more intelligent do consider the personality items as more meaningful and give more extreme responses, as suggested by Austin et al. (2000), then intelligence could possibly predict the degree to which individuals ponder their responses to personality items and/or respond to subtle item nuances. It should be noted that a research project utilizing intelligence to predict the depth of self-reflection in responding to personality items may need to control for verbal intelligence as verbal intelligence is linked to word generations (Harris 2004).

## 5. Limitations

Although the data represent a real-life situation, the results may have been slightly restricted because of the high-stakes situation. As the participants were applicants, they may have modulated their responses to the personality items to present themselves in a more favourable manner. The fact that all the participants were candidates for managerial positions in a forestry products company may have reduced the range of personality scores. This restriction may also apply to the sample’s level of intelligence. Future studies may try to test the differentiation of personality by intelligence in samples with a more heterogeneous intelligence distribution and go beyond high vs. low comparisons. Future studies may also go beyond testing measurement invariance across groups, to identify homogenous populations within the range of intelligence by, for example, mixed Rasch models. The data may also have been restricted as the sample was almost entirely men. A more balanced sample is needed in future studies. The final limitation, as stated above, was that item data were not available. Future studies may wish to examine concepts such as person reliability (Escorial et al. 2019) or moderated factor models at the item level (Murray et al. 2016). Future studies may also wish to examine how social desirability and/or impression management, in addition to intelligence, may influence personality item responses.

## 6. Conclusions

The present study provides little support for the differentiation of personality by intelligence hypothesis. In particular, the results suggest that there is no greater variability in the scale scores of the 16PF in applicants scoring higher in intelligence. Therefore, for applicants, it can be suggested that the factorial structure of the 16PF will most likely remain the same across groups varying in intelligence. Additional research is required in order to determine whether the hypothesis is generally less applicable in high-stakes personality-assessment scenarios such as the current one, which are becoming extremely common in pre-hire testing situations (Kantrowitz et al. 2018). Moreover, the fact that the differentiation of personality by intelligence has now received only weak support in the current study and even less in Murray et al.’s (2016), both of which relied upon the 16PF, suggests that the particular personality scale that was used might play a role in differentiating personality as a function of intelligence. We speculate that the inductive scale-development strategy underlying the 16PF could be what sets it apart from other personality inventories, such as the PRF (Jackson 1989), with regards to differentiation by intelligence. If Cattell et al.’s (1970) inferences that inductively derived scales reflect the “true” structure of personality are correct, perhaps a higher degree of inter-trait correlational stability across levels of intelligence will be provided.

## Figures and Tables

**Table 1:** Variance and mean difference tests for the 16PF scales between the lower and higher intelligence halves.

Scale	Lower Intelligence Half	Higher Intelligence Half	*F*	*t*
	Mean (*SD*)	Mean (*SD*)		
Warmth	5.56 (2.02)	5.35 (2.00)	0.36	1.60
Reasoning	7.28 (1.75)	8.14 (1.71)	0.40	−7.94 *
Emotional Stability	6.07 (1.65)	6.25 (1.59)	0.44	−1.68
Dominance	6.37 (2.01)	6.46 (1.88)	1.72	−0.76
Liveliness	6.24 (1.79)	6.46 (1.80)	0.48	−1.87
Rule Conscious	6.62 (1.72)	6.16 (1.66)	4.11	4.31 *
Social Boldness	6.16 (1.66)	6.53 (1.95)	0.68	0.65
Sensitive	4.76 (2.08)	4.64 (2.13)	0.52	0.96
Vigilance	4.75 (1.81)	4.65 (1.73)	1.19	0.90
Abstractness	5.36 (1.84)	5.79 (1.82)	1.07	−3.76 *
Privateness	4.77 (1.97)	4.33 (2.03)	0.87	3.51 *
Apprehension	4.50 (1.53)	4.38 (1.54)	0.02	1.20
Open to Change	5.42 (2.01)	6.07 (2.04)	0.80	−5.05 *
Self-reliance	5.25 (2.06)	5.75 (2.01)	0.30	−3.90 *
Perfection	6.99 (1.77)	6.81 (1.72)	0.01	1.56
Tension	4.98 (1.88)	5.06 (1.75)	1.10	−0.70

* *p* < 0.01, two-tailed.

**Table 2:** Pattern loadings for the five-factor solution for the lower and higher intelligence groups following oblimin rotation.

Scale	Factor I		Factor II		Factor III		Factor IV		Factor V	
	Lower	Higher	Lower	Higher	Lower	Higher	Lower	Higher	Lower	Higher
Warmth	0.50	0.52	−0.05	−0.01	0.02	0.05	0.06	0.02	0.00	0.00
Reasoning	−0.07	−0.09	−0.13	−0.06	0.07	0.02	0.17	−0.10	0.15	−0.15
Emotional Stability	0.06	−0.02	−0.79	−0.66	−0.18	−0.08	0.06	−0.14	−0.15	0.16
Dominance	0.15	0.10	0.02	−0.01	−0.02	−0.03	0.59	−0.60	−0.09	0.10
Liveliness	0.59	0.61	0.10	−0.02	0.08	0.02	0.05	−0.18	0.08	−0.07
Rule Conscious	0.03	0.18	0.10	0.15	0.53	0.48	0.03	0.09	−0.35	0.28
Social Boldness	0.59	0.56	−0.20	−0.25	0.05	0.01	0.22	−0.32	0.10	0.11
Sensitive	0.18	0.20	0.07	0.03	−0.07	−0.25	−0.28	0.21	0.20	−0.12
Vigilance	0.05	0.05	0.43	0.28	−0.07	−0.07	0.19	−0.20	−0.08	0.07
Abstractness	0.01	0.13	0.01	−0.01	−0.08	−0.10	0.04	0.06	0.46	−0.64
Privateness	−0.14	−0.18	−0.01	−0.02	−0.04	−0.12	−0.40	0.41	−0.17	0.25
Apprehension	−0.09	−0.09	0.47	0.54	−0.06	−0.05	−0.17	0.07	−0.09	0.13
Open to Change	0.03	0.01	0.02	−0.11	−0.04	−0.09	0.42	−0.44	0.06	−0.11
Self-reliance	−0.52	−0.36	0.13	0.13	0.09	0.12	0.08	−0.06	0.30	−0.43
Perfection	0.06	0.04	−0.05	−0.12	0.64	0.66	−0.06	0.07	0.05	−0.16
Tension	−0.08	−0.10	0.66	0.7	−0.04	−0.01	−0.02	−0.02	0.08	−0.06
Sum of squared loadings	1.32	1.25	1.57	1.43	0.77	0.79	0.93	1.00	0.59	0.89
